# Molecular cytogenetics for a wheat–*Aegilops geniculata* 3M^g^ alien addition line with resistance to stripe rust and powdery mildew

**DOI:** 10.1186/s12870-021-03360-4

**Published:** 2021-12-06

**Authors:** Yongfu Wang, Xiaofang Cheng, Xiaoying Yang, Changyou Wang, Hong Zhang, Pingchuan Deng, Xinlun Liu, Chunhuan Chen, Wanquan Ji, Yajuan Wang

**Affiliations:** 1 College of Agronomy, Northwest A&F University, Yangling, 712100 China; 2 State Key Laboratory of Crop Stress Biology for Arid Areas, Yangling, 712100 China; 3 Shaanxi Research Station of Crop Gene Resources and Germplasm Enhancement, Ministry of Agriculture, Yangling, 712100 China

**Keywords:** *Aegilops geniculata* Roth, Molecular cytogenetics, SLAF-seq, FISH-GISH, Powdery mildew, Stripe rust

## Abstract

**Background:**

*Aegilops geniculata* Roth is closely related to common wheat (*Triticum aestivum* L.) and is a valuable genetic resource for improvement of wheat.

**Results:**

In this study, the W19513 line was derived from the BC_1_F_10_ progeny of a cross between wheat ‘Chinese Spring’ and *Ae. geniculata* SY159. Cytological examination showed that W19513 contained 44 chromosomes. Twenty-two bivalents were formed at the first meiotic metaphase I in the pollen mother cellsand the chromosomes were evenly distributed to opposite poles at meiotic anaphase I. Genomic in situ hybridization demonstrated that W19513 carried a pair of alien chromosomes from the M genome. Fluorescence in situ hybridization confirmed detection of variation in chromosomes 4A and 6B. Functional molecular marker analysis using expressed sequence tag–sequence-tagged site and PCR-based landmark unique gene primers revealed that the alien gene belonged to the third homologous group. The marker analysis confirmed that the alien chromosome pair was 3M^g^. In addition, to further explore the molecular marker specificity of chromosome 3M^g^, based on the specific locus amplified fragment sequencing technique, molecular markers specific for W19513 were developed with efficiencies of up to 47.66%. The W19513 line was inoculated with the physiological race E09 of powdery mildew (*Blumeria graminis* f. sp. *tritici*) at the seedling stage and showed moderate resistance. Field inoculation with a mixture of the races CYR31, CYR32, CYR33, and CYR34 of the stripe rust fungus (*Puccinia striiformis* f. sp*. triticii*) revealed that the line W19513 showed strong resistance.

**Conclusions:**

This study provides a foundation for use of the line W19513 in future genetic research and wheat improvement.

## Background

Wheat (*Triticum aestivum* L.) is an important grain crop cultivated worldwide. The grains are a major source of starch, dietary fiber and nutrients and provides raw materials for industrial food production [1]. Among them, wheat is the main food source in many countries, which provides more than 20% of the energy and protein for human beings around the world [2]. China is the largest wheat producer and consumer in the world [3]. Continued population growth, serious environmental degradation, sharp reduction in the area of effective cultivated land and the ongoing conflict in resource demands all represent severe challenges to food production [4]. Therefore, wheat breeding faces important challenges globally and in China in the twenty-first century [5]. The loss of genetic diversity of cultivated wheat reduces the quality and yield of grains and increases vulnerability to biotic and abiotic environmental stresses [6]. Maintaining the sustainable production and stability of wheat grain yields is closely associated with China’s food security and socio-economic development strategies [7, 8]. Wild relatives of wheat (including wild and cultivated forms) are a reservoir for a large number of genes for desirable agronomic traits, and thus, for example, are an important source of disease resistance genes for cultivated wheat [9].

*Aegilops* is closely related and genetically similar to *Triticum* [10]. Almost all species of *Aegilops* are cross-compatible with wheat and, therefore, are an excellent genetic resource for improvement of wheat quality and other agronomic traits [11]. A large number of disomic addition lines (DALs) of wheat and its wild relatives were established by distant hybridization and chromosome engineering [12]. *Aegilops geniculata* Roth (2*n* = 4*x* = 28) is an annual self-pollinating allotetraploid species classified in *Aegilops* sect. *Aegilops*. Its chromosome configuration is U^g^U^g^M^g^M^g^ of which the U^g^ and M^g^ subgenomes are derived from *Ae. umbellulata* Zhuk. (UU, 2*n* = 2*x* = 14) [13] and *Ae. comosa* Sm. (MM, 2n = 2 *x* = 14), respectively [14, 15]. In the course of long-term evolution, *Ae. geniculata* has evolved a wealth of morphological variation and harbors a large number of stress-adaptation genes that confer traits such as disease and insect resistance, salt tolerance [16], cold and heat tolerance, and precocious maturity [17]. Therefore, *Ae. geniculata* is an important wild gene source for wheat improvement [18].

Powdery mildew of wheat, caused by *Blumeria graminis* f. sp. *tritici*, is a serious airborne leaf disease worldwide [10, 19], and mainly occurs in cool areas with a maritime climate. Stripe rust is a recurring disease caused by the fungus *Puccinia striiformis* f. sp. *tritici* (*Pst*) [20] and, together with powdery mildew, causes massive losses to agricultural production [21]. Breeding and planting of resistant cultivars is the most economical, effective, safe and reliable means to control wheat diseases. Identification of disease-resistant germplasm is not only beneficial for the breeding of disease-resistant cultivars, but also broadens the diversity of disease-resistance genes [22]. The screening of germplasm for identification of sources of disease resistance is a common focus of research into disease resistance. Disease-resistance genes (*Yr8*, *Lr9*, and *Pm29*) have been transferred from the U and M genomes of *Aegilops* to common wheat [23–25]. The use of wild relatives of wheat to generate, screen, and identify novel genetic lines carrying disease-resistance genes [26] can lay a foundation for the transfer of additional disease-resistance genes to wheat.

Next-generation sequencing technology enables large-scale application of molecular and genome technology for crop improvement, and is important in the development of molecular markers, including markers for RNA-sequencing and other technologies [27, 28], such as whole-genome sequencing(WGS), genotyping-by-sequencing (GBS) and specific-locus amplified fragment sequencing (SLAF-seq) [29–31]. SLAF-seq is a method of simplified genome sequencing intended for large-scale genotyping, which has the advantages of long effective reads, high throughput, and flexible scheme design. In addition, SLAF-seq plays a fundamental role in molecular breeding and, for example, has been used to develop specific markers on chromosome 7E of *Thinopyrum elongatum* with high efficiency of up to 65.9% [32]. To date, most of the specific markers for *Aegilops* were derived from wheat markers, which were inefficiently developed. New markers need to be developed urgently to accelerate the process of theoretical research and gene editing for utilization in wheat breeding. The success of SLAF-seq in the development of chromosome-specific molecular markers provides strong technical support for rapid screening of exogenous materials and shortening of the breeding cycle.

In previous research done by our research group, ‘Chinese Spring’ (CS) was used as the female parent that was crossed with *Ae. geniculata* ‘SY159’ as the male parent. The F_1_ progeny were backcrossed with CS and repeated selfing was performed to generate the BC_1_F_10_ population. In the present study, molecular cytological analysis, in situ hybridization, functional molecular markers, development-specific molecular markers based on SLAF-seq, assessment of morphological traits and stress resistance were employed to genetically characterize the disomic addition line W19513 from this population. We identified the configuration and number of chromosomes as well as the homologous group relationship of the alien chromosomes, and confirmed the desirable disease resistance and agronomic traits of the line. The results provide a foundation for use of the W19513 line in future genetic research and breeding improvement.

## Results

### Cytological observations

**Fig. 1:**
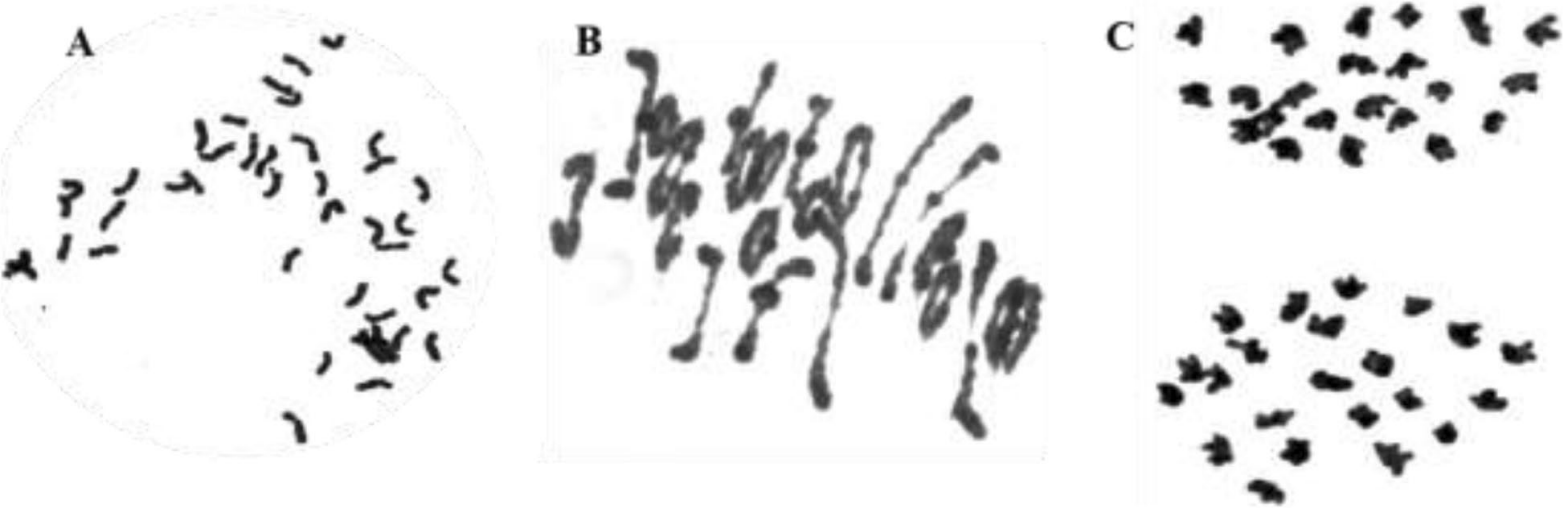
Root tip and meiotic analysis of chromosome characteristics of W19513, 2n = 22I + 22I Root tip cells at mitotic metaphase (**A**). Pollen mother cell chromosomal configurations at meiotic metaphase I, 2n = 44 (**B**). Pollen mother cell chromosomal configurations at prophase II, n = I (**C**)

The W19513 line was derived from the cross between CS and SY159, followed by one backcross to CS and repeated selfing to generate the BC_1_F_10_ population. Cytological examination revealed that the number of mitotic metaphase chromosomes (44) in the root tip of indoor- and field-cultivated plants was identical (Fig. 1A). Twenty-two pairs of chromosomes were observed in the pollen mother cells at meiosis, the chromosomes were evenly paired, and no unpaired chromosomes were observed (Fig. 1B). At meiotic anaphase chromosome separation was uniform, the chromosomes moved to the opposite poles synchronously, and no laggard chromosomes were observed (Fig. 1C). Therefore, W19513 showed high cytological stability.

**Fig. 2:**
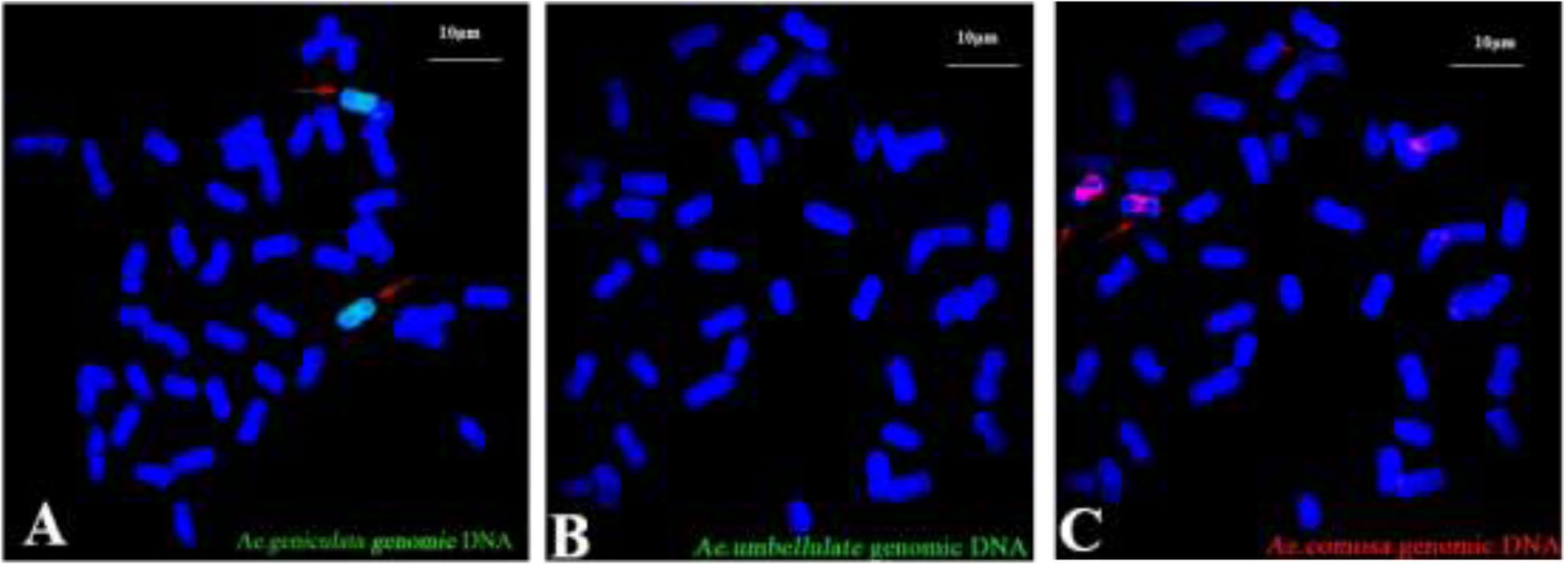
Karyotypes with the genomic composition variation of W19513, which was obtained by using GISH analysis. **A** SY159 genomic DNA. **B** The DNA of *Ae. umbellulata.* **C** The DNA of *Ae. comosa*. The red arrow indicates the introduction of alien chromosomes of *Ae. geniculate.* Chromosomes were counterstained by DAPI (blue). Bar indicates 10 μm

Genomic DNA of SY159 (as the probe DNA) and that of CS (as the blocking DNA) were extracted using a modified cetyltrimethylammonium bromide (CTAB) method [33] for genomic in situ hybridization (GISH). Two chromosomes showed the probe signal (Fig. 2A). This result implied that a pair of alien chromosomes were added to the genome of W19513. To further verify this result, GISH was conducted using CS as the blocking DNA, and *Ae. umbellulata* (Fig. 2B), and *Ae. comosa* **(**Fig. 2C) as the probe DNA. These results indicated that W19513 contained a pair of additional chromosomes from the M genome.

### Functional molecular markers analysis

**Fig. 3:**
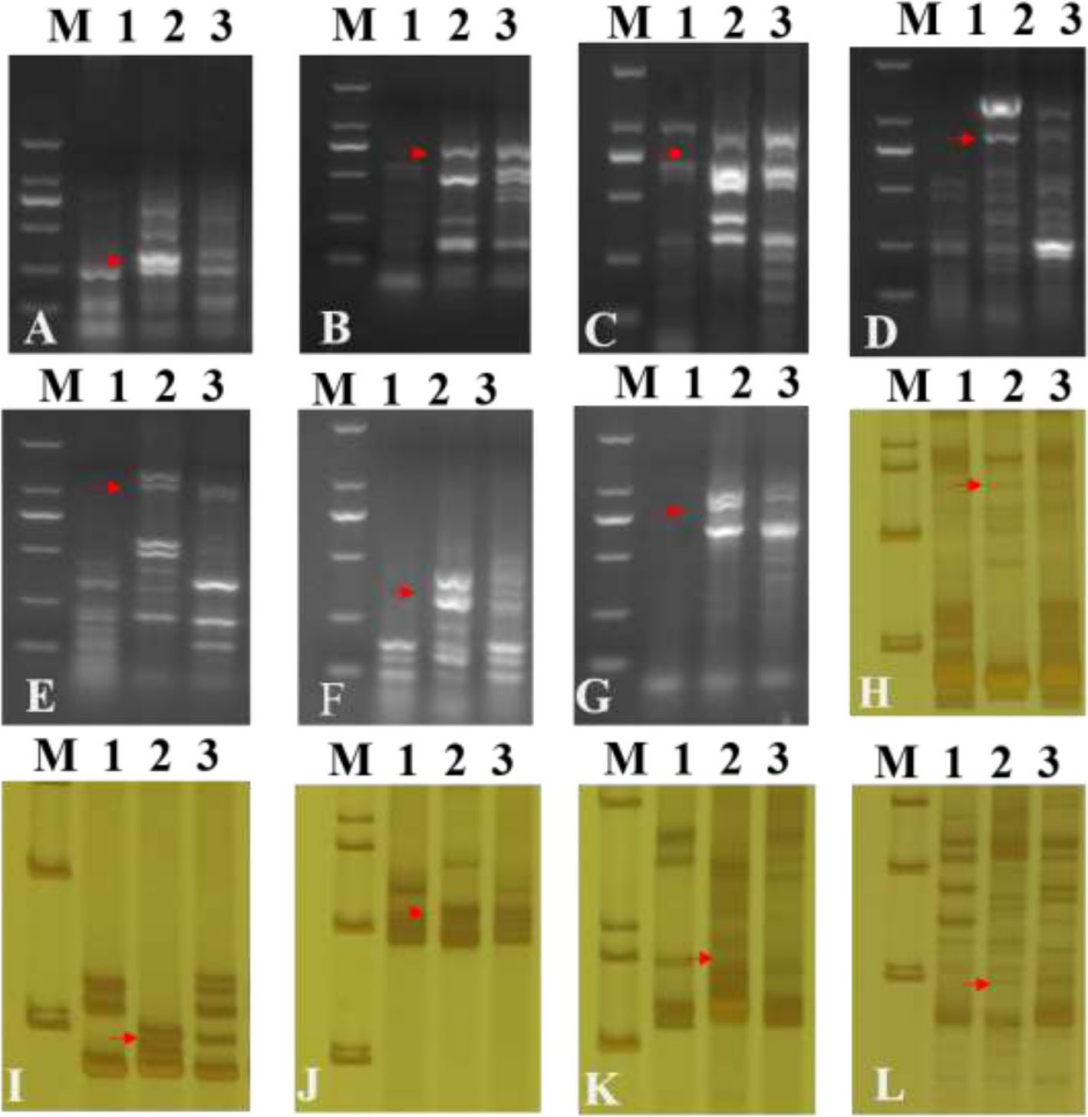
PLUG and EST–STS functional molecular marker analysis of W19513. (M). DL2000 (2 kb DNA ladder), (1). CS, (2). SY159, (3). W19513. (A-G). The PLUG markers amplification results with *TNAC*1364*, TNAC*1383*, TNAC*1300, *TNAC*1627, *TNAC*1364, *TNAC*1627, *TNAC*1341. **A**–**D**. The *TaqI* digestion; **E**–**G**. The *HAEII* digestion. **H**–**L**. The EST–STS markers amplification results with *BM134465*, *CD343475*, *BQ169491*, *CD452844*, *BF429203*. The red arrows indicate the SY159 specific bands

To determine the homologous group relationship of the alien chromosomes pair, 156 pairs of expressed sequence tag–sequence-tagged site (EST-STS) primers and 173 pairs of PCR-based landmark unique gene (PLUG) primers distributed across the seven homologous groups were analyzed using molecular markers. The pair of alien chromosomes was then compared with the amplified bands of the parents SY159 and CS. Five primers (*BM134465*, *CD343475*, *BQ169491*, *CD452844*, and *BF429203*) amplified specific bands (Fig. 3H–L). Specific bands of *Ae. geniculata* were amplified by five PLUG primers (*TNAC1300*, *TNAC1341*, *TNAC1383*, *TNAC1627*, and *TNAC1364*) (Fig. 3A–G), all of which belonged to the third homologous group (Table 1). Thus, the alien chromosomes belong to the third homologous group of the M genome (3 M).

**Table 1:** Expressed EST–STS and PLUG marker list W19513

Marker	Type	Primer (5′-3′)	Location	Geltype/Restrictionenzyme	Tm °C/t (h)
*TNAC*1300	PLUG	F: TCTGCAGGTTCGGTAGACAAT	3AS 3BS 3DS	2% agarose gel/*TaqI*	60
		R: AGTACGGGAGGACGCATGT			
*TNAC*1341	PLUG	F: GTTGAAGCCTACATGCCACAC	3AL 3BL 3DL	2% agarose gel/*TaqI*	54
		R: TAGCATGGGCTCCTAACATTG			
*TNAC*1383	PLUG	F: GCGGTCGATCTTCTTCAAGTC	3AS 3BS 3DS	2% agarose gel/*TaqI/HaeIII*	60
		R: TCAGATGGACTATGGGAGCAC			
*TNAC*1627	PLUG	F: CAGGAGGCCTACGAGACG	3AS 3BS 3DS	2% agarose gel/*TaqI*/*HaeIII*	60
		R: TTCTTCAGCTCGGATATTTGG			
*TNAC*1364	PLUG	F: CGTCAGGCTCAGGGTGTC	3AL 3BL 3DL	2% agarose gel/*TaqI*	60
		R: AAAGAGCCTCTGTCTCTCAGG			
*BM134465*	EST-SSR	F: CAATTGAACCCATCCGAAAG	3AL 3BL 3DL	8% non-denaturing	60
		R: CTGCCCGAACTATCCACAAT		polyacrylamide gel 8% non-denaturing/−	
*CD343475*	EST-SSR	F: CCGAAGACGAAGTCGAGAAC	3AS 3BS 3DS	8% non-denaturing	62
		R: ACACATCCCGTCCTTCTTTG		polyacrylamide gel 8% non-denaturing/−	
*BQ169491*	EST-SSR	F: TGGCCATCATTGAACTGAAA	3AL 3BL 3DL	8% non-denaturing	56
		R: CAATCAGATTTTTCGGCCAT		polyacrylamide gel 8% non-denaturing/−	
*CD452844*	EST-SSR	F: AAAAGTTGGCCACCAATCTG	3AL 3BL 3DL	8% non-denaturing	60
		R: TGGCATATGTCGCTCTGAAG		polyacrylamide gel 8% non-denaturing/−	
*BF429203*	EST-SSR	F: CTTCGTAGCCTCCTCACTGG	3AL 3BL 3DL	8% non-denaturing	60
		R: AGATTATGTGCGTGCTGTGC		polyacrylamide gel 8% non-denaturing/−	

### In situ hybridization

**Fig. 4:**
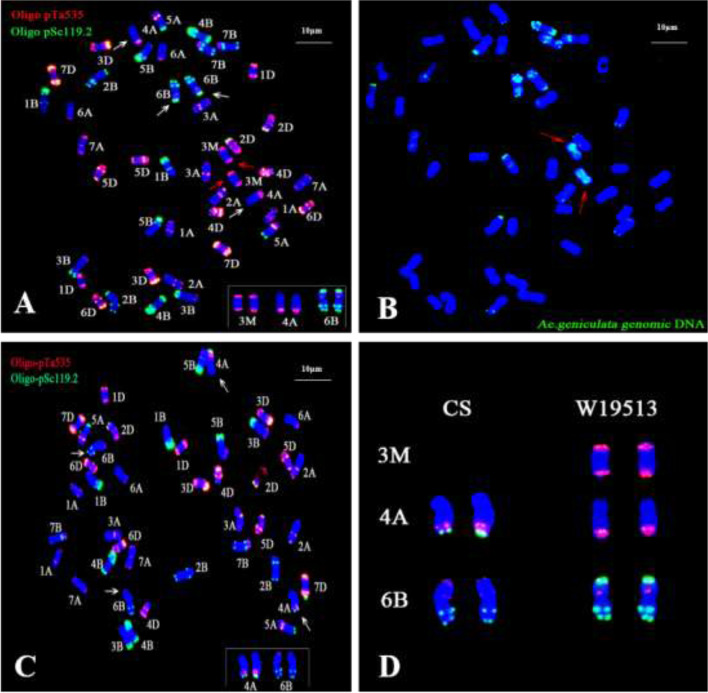
Karyotypes with the genomic composition variation of W19513, which was obtained by using FISH and sequential FISH-GISH analysis. FISH used Oligo-pSc119.2 (green) and Oligo-pTa535 (red) as probes. The SY159 genomic DNA was used as probe for sequential FISH- GISH. The red arrow indicates the introduction of alien chromosomes of SY159*.* White arrows indicated the structural variation of the chromosomes 4A and 6B. **A** FISH of W19513. **B** GISH of W19513 in the same cell. **C** Analysis of CS by FISH. **D** FISH signal comparison between CS and W19513 on chromosomes 4A, 6B. Chromosomes were counterstained by DAPI (blue). Bar indicates 10 μm

The oligonucleotide probes Oligo-PTa535 and Oligo-pSc119.2 were used for fluorescence in situ hybridization (FISH) analysis, and then the pair of alien chromosomes was compared with the CS standard karyotype map [34]. The karyotype of W19513 was essentially identical to that of CS, comprising 42 chromosomes with an additional pair of alien chromosomes (Fig. 4A). The results of sequential FISH-GISH analysis further confirmed that the line W19513 carried a pair of alien chromosomes from *Ae. geniculata.* Comparison of the extra pair of chromosomes on the FISH map showed signal from the *Ae. geniculata* probe (Fig. 4B). Interestingly, comparison of the FISH karyotype of W19513 with that of CS (Fig. 4C) revealed variations in chromosomes 4A and 6B (Fig. 4D). Collectively, the aforementioned results showed that W19513 carried an additional pair of chromosomes 3M^g^ derived from *Ae. geniculata.*

### Development of specific molecular markers

**Fig. 5:**
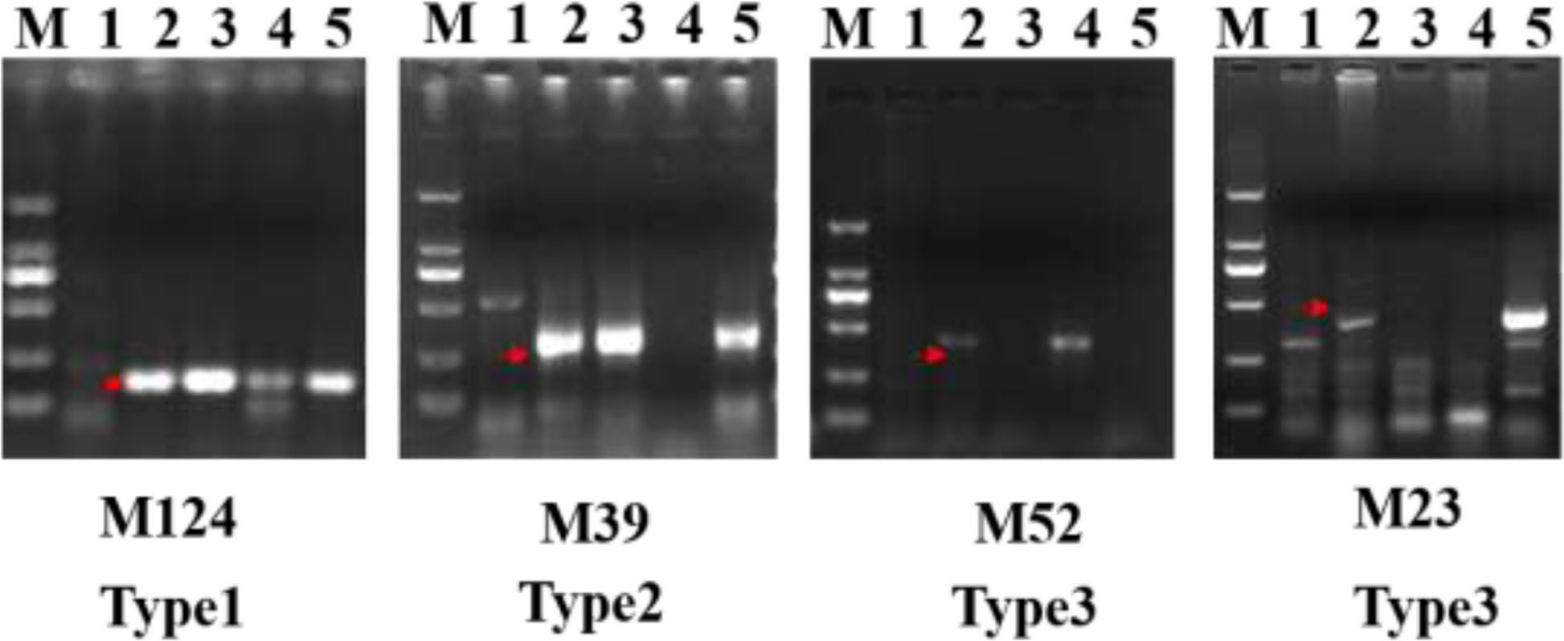
Amplification patterns of *Aegilops* accessions using W19513. SLAF markers M124, M39, M52 and M23. (M) DL2000 (2 kb DNA ladder). (1) CS. (2) SY159. (3) *Ae. comosa*. (4) *Ae. umbellulate*. (5) W19513. The red arrows indicate the specific bands

The SLAF-seq data comprised a total of 4,733,453, 8,752,434, and 6,943,541 raw reads for CS, SY159, and W19513, respectively. The average Q30 score was 94.15% and the average GC content was 48.66%. After filtering out low-depth data, the final numbers of SLAF-seq reads were 314,571, 193,701, and 371,647 for CS, SY159, and W19513, respectively, and the average sequencing depth was 6.3850. Using the Burrows–Wheeler Alignment (BWA) tool, 2888 reads were observed to show 50% similarity to the CS reference genome (IWGSC RefSeq v1.0). Among these reads, 634 reads showed at least 90% similarity to SY159 and were considered to be specific fragments of chromosome 3M^g^. To develop chromosome 3 M-specific markers, 128 primers were designed based on 128 randomly selected fragments and were used to amplify the sequence of CS, SY159, *Ae. comosa*, *Ae. umbellulata* and W19513. Among these markers, 61 markers amplified SY159-specific bands with a maximum success rate of 47.66% (Table 2). Ultimately, 61 sequences of chromosome 3 M were obtained (data not shown). The 61 3 M-specific markers were further divided into three categories, namely W19513, SY159 and *Ae. comosa* and *Ae. umbellulata*, of which four markers (Fig. 5) showed the same amplification patterns in W19513, SY159, *Ae. comosa* and *Ae. umbellulata* (Fig. 5**Type 1**); 13 markers amplified the same bands in W19513 and SY159, but not the corresponding bands in *Ae. comosa* and *Ae. umbellulata* (Fig. 5**Type 2**); and 44 markers amplified the same bands in W19513, SY159, and *Ae. comosa*, but not *Ae. umbellulata* (Fig. 5**Type 3**).

**Table 2:** Detection of W19513 specific-locus amplified fragment sequencing (SLAF) markers

Type	SY159	*Ae. Comosa*	*Ae.Umbellulata*	W19513	No. of Specific
	(UUMM)	(MM)	(UU)		
Type1	+	+	+	+	4
Type2	+	+	–	+	44
Type3	+	–	–	+	13

### Assessment of agronomic traits and disease resistance

**Fig. 6:**
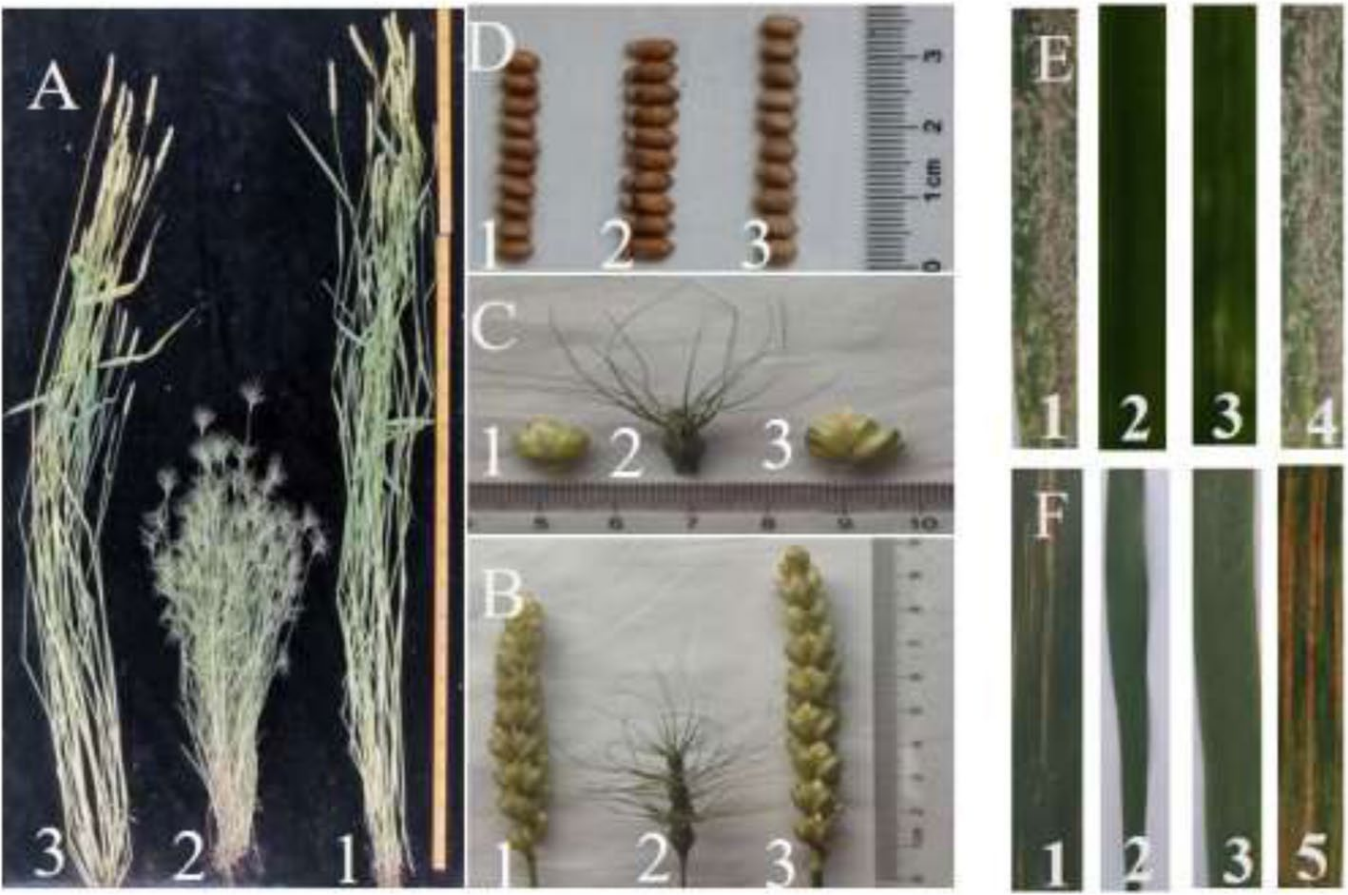
Morphological characteristics (**A**-**D**), Powdery mildew (**E**) and Stripe rust reaction (**F**) of W19513. (1) CS; (2) SY159; (3) W19513; (4) Shaanyou225; (5) Xuixianhong (**A**) plants. (**B**) spikes. (**C**) florets. (**D**) grain width. (**E**) Identification of resistance to powdery mildew at seedling stage. (**F**) Symptoms in response to inoculation with the mixture of *Pst* races at the adult stages

The agronomic traits of W19513 and the parents CS and SY159 were analyzed. No significant differences in plant height, number of grains per spikelet, spikelet number, spikelet length, and 1000-grain weight were observed between W19513 and CS. However, notable differences in the lower spike nodes between W19513 and CS were noted. Significant differences were observed in plant height, spikelet number, tiller number, and 1000-grain weight between W19513 and SY159. These results inferred that the W19513 line showed overall morphological intermediary between the parents and produced long panicle nodes and stable agronomic traits (Fig. 6A–D, Table 3).

**Table 3:** Analysis of the agronomic traits of W19513 and its parents (CS, SY159)

Materials	Plant	Tillering	Spikelets/	Kernels/	Spike	Sub panicle	Thousand Kernel	Awnedness
	Height (cm)		Spike	Spikelet	Length(cm)	node(cm)	Weight(g)	
CS	128	21	21	4	9	40	32	Awn less
SY159	59	68	19	2	5	23	23	Long awn
w19513	138	33	20	4	9	47	34	Awn less

The degree of resistance to powdery mildew was determined by inoculation with powdery mildew physiological race E09 at the seedling stage (Fig. 6E). In a bonsai experiment in an artificial climate box, the mortality of CS and Shanyou 225 (the susceptible control) was scored as 4. The resistance of *Ae. geniculata* was scored as 0, which indicated immunity. The W19513 line showed moderate resistance. In a field experiment to assess resistance to stripe rust, four physiological races (CYR31, CYR32, CYR33, and CYR34) of stripe rust were mixed with talc and used as a powder to inoculate plants. Huixianhong (HXH) was used as the susceptible control. The resistance of *Ae. geniculata* was scored as 0, which thus indicated immunity. The mortality of HXH and CS was scored as 4; the resistance type of the W19513 line was scored as 1, which thus indicated higherresistance (Fig. 6F). These results indicated that the resistance gene in the W19513 line was derived from SY159.

## Discussion

Since the founding of the People’s Republic of China in 1949, substantial progress has been achieved in increasing wheat production. From 1949 to 2000, the average wheat yield per hectare has increased from 0.70 tons to 3.86 tons [35]. The International Maize and Wheat Improvement Centre (CIMMYT) views the development of hybrid cultivars as a promising means to boost crop yields because of the growing global demand for wheat [36]. Hybrids can be grown in marginal environments and at low sowing densities because the plants are more vigorous, robust, and stress resistant than pure-line cultivars under marginal conditions [37]. Alien chromosomes, which carry potentially useful agronomic traits, have been introduced into the common background of wheat and have become a useful resource for wheat breeding. In addition, disomic lines are an important bridge for the introduction of desirable alien genes into common wheat [38–40].

*Ae. geniculata* shows excellent resistance to stress and introduction of its favorable traits into common wheat will improve wheat yield and quality. *Aegilops* and *Triticum* are closely related genera and show strong genetic similarities [10]. The method of C-banding analysis [41] is used to identify the chromosome of the whole set of *Aegilops* and its telomeres that are added to the common wheat [42]. Therefore, the transfer of genes from *Aegilops* species to common wheat can be achieved readily by hybridization. Chromosome 7M^g^ of SY159 was incorporated as a disomic addition and substitution line in common wheat, which not only enhanced wheat resistance to powdery mildew, but also increased the 1000-grain weight [6, 43]. In practical application, many disease-resistance genes are transferred by the introduction of alien chromosomes, which plays an important role in genetic breeding. Previous experiments have shown that the stripe rust resistance gene *Yr40* and the leaf rust resistance gene *Lr57* were both derived from chromosome 5M^g^ of *Ae. geniculata* [44]. Similarly, the powdery mildew resistance gene *pm29* located on chromosome 7D was derived from *Ae. geniculata* [25]. In the present experiment, assessment of agronomic traits demonstrated that the line W19513 is genetically stable and morphologically intermediate between the parents CS and SY159. The yield of the W19513 line was higher than that of the parents. In addition, the desirable panicle traits are important for improvement of wheat yield, which was previously achieved through the introduction of chromosomes from wild relatives of wheat [45]. With regard to disease resistance, the line W19513 was moderately resistant to race E09 of powdery mildew and highly resistant to four stripe rust races in the field **(**Fig. 6E, F**)**. These favorable characters are suitable for introduction into wheat. The W19513 line can be used as a donor source to introduce new genes in wheat breeding. In addition, wheat breeding must coordinate the interaction between yield and yield components, which is of great importance for future wheat production and breeding.

Geneticists and an increasing number of plant breeders consider that molecular marker-assisted selection is a valuable tool for germplasm selection to optimize selection efficiency in plant breeding programs [46–48]. Molecular markers are based on nucleotide sequence variation between individuals and thus are a direct reflection of genetic polymorphism at the DNA level. Analysis of wheat–*Aegilops* derivative lines using EST-STS and PLUG molecular markers can distinguish homologous groups of heterogeneous chromosomes and track exogenous chromosomes [49]. In the present study, using these functional molecular markers, we quickly detected that the alien chromosomes belonged to the third homologous group, thus reducing detection time and cost **(**Fig. 3**)**. At present, the development efficiency of conventional specific markers is low, the cost is high, and an alien chromosome cannot be quickly screened, which causes great inconvenience in breeding [50]. SLAF-seq technology exhibits a high success rate, high specificity and stabilityand low cost for the development of plant chromosome-specific molecular markers. For example, based on SLAF-seq technology, Bourtzis et al. [32] randomly developed 135 pairs of primers from chromosome 7E in *Th. elongatum* with efficiency of 65.9%. Markers specific to *Aegilops biuncialis* were developed with a success rate of up to 40.33% [51]. The success rate of 1JS-specific marker development from an alien chromosome of *Th. ponticum* was 52.98% [52]. The development of SLAF-seq technology has certain practical importance [32]. In the current study, 634 fragments specific for the 3 M chromosome of SY159 were obtained using SLAF-seq technology based on 128 randomly selected fragments. Sixty-one pairs of molecular markers were screened with an effective rate of 47.66%. The development of 3M^g^-specific markers with SLAF-seq technology was beneficial for identification of the disomic addition line derived from *Ae. geniculata* and the development of related probes, which lays a foundation for development of a karyotype map of *Ae. geniculata* in the future. Interestingly, by applying SLAF-seq technology to chromosome 3M^g^ of *Ae. biuncialis*, we observed that the same bands were detected in W19513, SY159, *Ae. comosa* and *Ae. umbellulata* but not CS **(**Fig. 5**)**. We hypothesized that the chromosomes of *Ae. comosa* and *Ae. umbellulata* were homologous. It will be extremely valuable to develop many chromosome-specific molecular markers for *Ae. geniculata*, not only to quickly identify an alien chromosome introduced into common wheat, but also to accelerate the exploration and utilization of its useful agronomic traits or disease resistance in practical wheat production.

FISH is a powerful tool for the identification of distant hybrids, and to elucidate the origin and evolution of allopolyploids [53]. Tang et al. [54] designed 120 oligonucleotide probes based on new tandem repeat sequences, 29 of which showed specific signals on wheat chromosomes. It was important to use new oligonucleotide probes to identify wheat chromosomes or specific segments of wheat chromosomes. We applied GISH technology as described by Shu-Lan et al. [55] with minor modifications. GISH proved that only one pair of alien chromosomes was introduced in the W19513 line and that the chromosomes belonged to the M genome. Further evidence showed the presence and signal morphology of the alien chromosomes. Furthermore, by combining molecular markers, we demonstrated that W19513 contained one additional pair of alien chromosomes designated 3M^g^. Chromosomal rearrangement plays an important role in plant evolution. Huang et al. [56] conducted a FISH analysis of CS and 373 Chinese wheat cultivars and observed that 14 structural rearrangements had occurred in 148 (39.7%) cultivars. Badaeva et al. [57] used the C-banding technique to show that chromosomal rearrangement had occurred in 208 tetraploid wheat,252 hexaploid wheat, and 39 triticale accessions. The introduction of alien chromosomes may lead to changes in the structure of chromosomes of common wheat and affect the expression of a suite of genes [52, 58, 59]. Przewieslik-Allen et al. [60] analyzed 471 materials and found that chromosome rearrangement plays an important role in the genetic diversity of varieties. In the present study, FISH analysis suggested that 38 chromosomes were consistent with the standard karyotype map of CS, and variation in 4A and 6B signals were observed. The green signal at the tip of the chromosome 4A long arm was highly weakened and a strong green signal was observed on the short arm of chromosome 6B. These signal changes may be caused by structural rearrangement of wheat chromosomes or polymorphism of FISH signals. It can be inferred from the aforementioned results that chromosomal rearrangement and polymorphism often occur in widely cultivated wheat cultivars and experimental breeding materials and are essential in breeding new cultivars. The introduction of alien chromosomes may also stabilize their structure through chromosomal rearrangement and polymorphism, which can be utilized in genetic breeding. However, the utilization of such rearrangements requires further exploration.

## Conclusions

In this research, W19513 was, derived from BC_1_F_10_ progeny of a cross between CS and SY159. This line was characterized by molecular cytological, in situ hybridization, functional molecular markers, development specific molecular markers based on SLAF-Seq, morphological and stress resistance identification. The results showed that line W19513 was a wheat– *Ae. geniculata* 3M^g^ alien addition line, with excellent endowing characteristics. The line with resistance genes was created, screened and identified by using wild related species of wheat, which could lay a foundation for further shifting of resistance genes to wheat.

## Methods

### Plant materials

The wheat – *Ae*. *geniculata* disomic addition line W19513 was derived from a cross between common wheat ‘Chinese Spring’ (AABBDD, 2*n* = 42) and *Ae. geniculata* ‘SY159’ (UUMM, 2*n* = 28). The F_1_ progeny were backcrossed with CS, and then the progenies were repeatedly selfed to generate the BC_1_F_10_ population. Wheat ‘Shaanyou 225’ was used as the susceptible control for assessment of powdery mildew resistance and ‘Huixianhong’ was used as the susceptible control for assessment of stripe rust resistance. They were all preserved at the College of Agronomy, Northwest A&F University, Yangling, China. *Ae. geniculata* SY159, *Ae. umbellulata* (UU, 2*n* = 2*x* = 14), and *Ae. comosa* (MM, 2*n* = 2*x* = 14) were provided by Professor Lihui Li and Xinming Yang, Chinese Academy of Agricultural Sciences, Beijing, China.

### Cytogenetic analysis

The seeds were soaked in a petri dish with filter paper for one day. The water was poured from the petri dish and then the seeds were placed in an incubator in the dark. After the root length attained 2–3 cm, the root tip was excised and treated with nitrous oxide for ~ 2 h. The root tip was then fixed in 90% acetic acid, 70% ethanol was added, and the root tips were stored at− 20 °C. Following the method of Han et al. [61], the white film was made, and the chromosome number in the root tip cells was observed. In accordance with the method of Wang et al. [43], a typical anther was fixed with Carnoy’s reagent (ethanol:chloroform:acetic acid, 6:3:1) for 24 h prepared in 1% magenta acetate. A fluorescence microscope (BX53, Olympus, Tokyo, Japan) and CCD imaging system were used to observe and photograph mitosis in the root tip cells and meiosis in the pollen mother cells.

### FISH, GISH, and sequential FISH–GISH

Following the method of Tang et al. [34], the oligonucleotide probes Oligo-PTa535 (red) and Oligo-pSc119.2 (green) were used to perform FISH. The signal was compared with the existing karyotype map for CS to identify the alien chromosomes. For sequential FISH–GISH analysis, the film prepared for FISH were soaked in alcohol for 2–3 days and then naturally dried. For GISH, genomic DNA of *Ae. geniculata* was used as a probe and mixed with different concentrations of CS genomic DNA as the blocking DNA. Using a ratio of 1:300, the probe and blocking DNA were hybridized at 56 °C. The fluorescence signal from the chromosomes was observed with a fluorescence microscope (Olympus BX-53). By combining the results from these two techniques, variation in the alien chromosomes could be further determined. To aid with chromosome observation, the chromosomes were stained with the blue-fluorescent DNA stain 4′,6-diamidino-2-phenylindole (DAPI).

### Functional molecular markers analysis

Molecular markers are powerful tools for detection of alien chromosomes or fragments [62]. Genomic DNA of all plant materials was extracted using a modified CTAB method [33]. The molecular markers used for homologous group screening were amplified using EST-STS and PLUG primers selected from among the PCR Primers for Grain Genes (http: //wheat.pw.usda.gov/SNP/new/pcr_primers.shtml). The homologous group to which the alien chromosomes belonged was preliminarily determined using this method. The specificity and homoeologous group of the EST-STS primers was assessed by differential separation of the products by polyacrylamide gel electrophoresis according to the DNA size. The PLUG markers were separated by 2% agarose gel electrophoresis in 1% TAE buffer solution; these markers not only showed a high level of polymorphism, but also detected variation and homologous chromosome groups.

### Development of specific molecular markers

The SLAF-seq method is a simplified genome sequencing technology independently developed by Biomarker [63] and shows three distinguishing characteristics: (1) deep sequencing to ensure genotyping accuracy, (2) a reduced representation strategy to reduce sequencing costs, and (3) a predesigned reduced representation scheme to optimize marker efficiency [31]. To increase marker specificity and efficiency, sequences that showed 50% similarity to CS (http://www.wheatgenome.org/News/Latest-news/IWGSC-Reference-Sequence-v1.0-browser-now-available-at-URGI) were selected using the BWA tool [51]. From these markers, sequences that showed at least 90% similarity to SY159 were selected. After PCR amplification, specific bands were detected in 1% agarose gel [63]. All primers were synthesized by the Beijing Aoke Ding Sheng Biotechnology Co., Ltd. (Beijing, China).

### Assessment of agronomic traits and disease resistance

The plant materials were grown at the Northwest A&F University in late September 2019. The row length was 1.0 m, the row spacing was 0.25 m, and the spacing between plants was 5–10 cm. Ten plants were randomly selected at harvesting in 2020 to compare the agronomic traits of W19513, CS, and SY159. In the field, following the assessment method of Wang et al. [43], we recorded plant height, tiller number, spike length, spikelet number, grain number per spikelet, and spikelet node characters, and the 1000-grain weight and grain width were measured after maturity.

The degree of resistance to powdery mildew was assessed at the seedling stage. Ten plants were planted in each pot with three replicates. Shaanyou 225 was used as the susceptible control. At the two-leaf stage, the plants were inoculated with *B. graminis* f. sp. *tritici* physiological race E09 by artificial shaking. About two weeks after inoculation, the resistance to E09 was evaluated in accordance with the method of Wang et al. [64] using a 0–4 scale, 0 = immunity, 0; = near immunity, 1 =  highly resistant, 2 = moderately resistant, 3 = moderately susceptible, and 4 = highly susceptible. The assessment of resistance to stripe rust in the field was conducted in the experimental field of the College of Agronomy, Northwest A&F University. The physiological races CYR31, CYR32, CYR33, and CYR34 were mixed in equal proportions with talc 1:1:1:1. The mixture of strains was used to inoculate plants by shaking the powder onto the leaves. HXH was used as the susceptible control. The reaction type was recorded in accordance with the standard 0–4 scale [65].

## Data Availability

All data and materials are available in the article.

## Abbreviations

*Pst*: *Puccinia striiformis* f. sp. *Tritici*
SLAF-seq: specific-locus amplified fragment sequencing
CS: Chinese Spring
*Ae. geniculate*: *Aegilops geniculate*
CTAB: cetyltrimethylammonium bromide
GISH: genomic in situ hybridization
EST-STS: expressed sequence tag–sequence-tagged site
PLUG: PCR-based landmark unique gene
BWA: Burrows–Wheeler Alignment
FISH: fluorescence in situ hybridization
HXH: Huixianhong
CIMMYT: International Maize and Wheat Improvement Centre
DAPI: 4′,6-diamidino-2-phenylindole.
